# Edema in Pregnancy: A Common yet Understudied Maternal Concern

**DOI:** 10.7759/cureus.103915

**Published:** 2026-02-19

**Authors:** Tejal Shelat, Khyati H Patel, Nandita Ganne, Alyssa Polk, Erica Zell, Stephen K Stacey

**Affiliations:** 1 Family Medicine, Mayo Clinic Health System, La Crosse, USA

**Keywords:** epidemiology, lower extremity edema, maternal comorbidities, obesity in pregnancy, postpartum, risk factors for edema

## Abstract

Objective: This study aims to assess the prevalence of lower-extremity edema among postpartum patients and identify potential associated risk factors.

Methods: A survey evaluating the presence of lower-extremity edema and potential risk factors was conducted among 52 patients across two hospitals in La Crosse, Wisconsin, and Mankato, Minnesota, for six months between August 1, 2022, and January 31, 2023. All participants were informed of their rights and provided written consent. All adult postpartum women were screened for inclusion. Exclusion criteria included communication barriers, active cancer, deep vein thrombosis, infection involving the pelvis or lower extremities, recent lower-extremity fractures, recent non-obstetric surgery, or current participation in experimental treatments. Data were collected using a paper-based survey. Logistic regression analysis was performed using BlueSky statistical software (BlueSky Statistics LLC, Chicago, IL, USA) to examine the relationship between edema and potential risk factors, including delivery type, pregnancy complications, and comorbid conditions such as obesity and chronic diabetes. Models were constructed with the dependent variable (presence of edema) regressed on selected independent variables (risk factors).

Results: Of the 52 postpartum participants, 18 (34.6%) reported experiencing edema of pregnancy (EOP). Among them, 13 (76.5%) reported edema during both pregnancy and the postpartum period, 4 (23.5%) reported edema only during pregnancy, and 1 (5.9%) reported postpartum-only edema. Obesity emerged as a statistically significant risk factor for the development of EOP (p < 0.05).

Conclusion: This study identified obesity as a statistically significant risk factor for EOP. Although several other factors did not reach statistical significance, their potential clinical relevance warrants further investigation. Future large-scale, longitudinal studies are needed to better understand the multifactorial influences of maternal characteristics and comorbidities on the development of pregnancy-related edema.

## Introduction

Edema of pregnancy (EOP) is an underrecognized yet highly prevalent condition that can cause significant physical discomfort, impair mobility, disrupt sleep, and provoke anxiety about potential complications such as preeclampsia or venous thromboembolism. It is often dismissed as a benign, self-limited phenomenon despite its considerable impact on maternal quality of life and the clinical uncertainty it generates. Surprisingly, despite its frequent clinical appearance, robust data regarding the prevalence, severity, and optimal management of EOP remain limited [1,2]. This knowledge gap leaves clinicians without clear guidance and patients without validation or effective symptom relief. A better understanding of the true prevalence of EOP and the populations most affected could lay the groundwork for more systematic evaluation, improved patient counseling, and more equitable care.

EOP manifests primarily in the lower extremities during either pregnancy or the postpartum period. The pathophysiology of EOP is multifactorial. Proposed mechanisms include compression of the inferior vena cava and iliac veins by the gravid uterus, nitric oxide-mediated vasodilation, and increased hydrostatic pressure secondary to elevated levels of corticotropin and estrogen [3]. Additionally, estrogen- and progesterone-induced venous wall distensibility contributes to venous valve incompetence, particularly during the second and third trimesters [4]. These factors, combined with reduced colloid oncotic pressure and diminished lymphatic drainage, occur naturally during pregnancy [5,6].

Established risk factors for EOP include gestational hypertension, pre-eclampsia, venous insufficiency, obesity, physical inactivity, prior deep venous thrombosis (DVT), and malignancy [7]. Early in pregnancy, venous insufficiency is typically compensated by lymphatic drainage; however, by the third trimester, lymphatic overload can result in combined lymphatic and venous insufficiency [7].

EOP has also been associated with several comorbid conditions, including preeclampsia, HELLP syndrome (hemolysis, elevated liver enzymes, low platelet count), gestational hypertension, DVT, chronic venous insufficiency, peripartum cardiomyopathy, intrahepatic cholestasis of pregnancy, hypothyroidism during pregnancy, nephrotic syndrome, and hepatic diseases such as Wilson’s disease [7]. It is unknown whether any of these associations and comorbidities are causally linked.

Various non-pharmacologic interventions have demonstrated efficacy in alleviating EOP. Ochalek et al. highlighted the benefits of compression stockings and regular physical activity in reducing lower limb edema [7]. Similarly, Coban et al. reported a statistically significant reduction in lower-leg edema following foot massages in women beyond 30 weeks' gestation without comorbidities such as preeclampsia or systemic diseases [8]. Water immersion therapy has also shown promise in mitigating EOP symptoms [9,10].

Previous studies estimate the prevalence of EOP at approximately 66-67%; however, these studies have limitations in accurately defining its true prevalence due to being completed within a limited geographical area and a lack of generalizability [11,12]. There is a lack of current literature on the prevalence of lower-extremity edema during the intra- and postpartum periods, particularly within the United States. Prior studies have primarily been conducted in international settings, limiting their generalizability to the U.S. population. To better characterize the current prevalence of EOP in our patient population, we administered a targeted survey to postpartum women during their hospital stay following childbirth. Our study aimed not only to estimate prevalence but also to explore potential risk factors and associated conditions, providing a more comprehensive understanding of this clinical concern.

## Materials and methods

Human subjects were involved in this study. The study was approved by Mayo Clinic’s Institutional Review Board to be conducted among postpartum patients admitted to the Mayo Clinic Health System facilities in La Crosse, Wisconsin, and Mankato, Minnesota, between August 1, 2022, and January 31, 2023. Participants were selected through convenience sampling and included all women admitted following any type of delivery within 24 hours after delivery. Exclusion criteria included communication barriers, active malignancy, diagnosed DVT, skin and soft tissue infections involving the pelvis or lower extremities, recent lower-extremity fractures, recent non-obstetric surgery, and current participation in other experimental treatment protocols.

Prior to enrollment, all patients provided verbal informed consent after being informed of their rights as study participants. Enrolled participants completed a structured questionnaire designed to assess the prevalence of lower-extremity edema during pregnancy or the postpartum period within this community. The questionnaire collected detailed information on potential risk factors contributing to edema, including maternal age, obstetric history, gestational age at delivery, mode of delivery, significant pregnancy-related complications, prior lower-extremity conditions, physical activity levels during pregnancy, and fluid intake (see Appendix 1). Additional data regarding the presence, severity, and treatments used for edema during pregnancy were also recorded. Resident physicians administering the questionnaire were available at all times to explain all terms and answer questions to the satisfaction of each participant. Multiple answers were possible for this question, and each was recorded as reported.

Statistical analysis was performed using BlueSky software (BlueSky Statistics LLC, Chicago, IL, USA). Logistic regression was employed to explore associations between the presence of edema (binary outcome) and individual risk factors. This approach was chosen for its capacity to model dichotomous dependent variables and to estimate odds ratios as measures of association. Separate univariate logistic regression models were constructed for each independent variable (risk factor) with edema as the dependent variable.

## Results

A total of 52 postpartum women participated in the study. Among them, 18 individuals (34.6%) reported experiencing EOP. Of these, 13 participants (76.5%) reported edema during both pregnancy and the postpartum period, four (23.5%) experienced edema exclusively during pregnancy, and one participant (5.9%) reported postpartum edema only (Table 1).

**Table 1:** Prevalence of edema in pregnancy

Type of Edema	Number (Percent)
Pregnancy and postpartum edema	13 (76.5%)
Pregnancy edema	4 (23.5%)
Postpartum edema	1 (5.9%)

The mean age of participants was 30 years (range 21-39), with an average parity of 2.1 children (range 1-6). The majority delivered between 35 and 41 weeks' gestation (Table 2). Regarding medical comorbidities, chronic hypertension was reported in two participants (3.8%), gestational hypertension in eight (15.4%), and preeclampsia in four (7.7%). Among those diagnosed with either chronic or gestational hypertension, five individuals (50%) reported EOP. Similarly, two of the four participants with pre-eclampsia (50%) reported edema (Table 2). Of the 11 participants who self-identified on the survey as obese, eight (72.7%) reported lower-extremity edema. Of the 41 non-obese participants, nine reported edema (22.0%) (Table 2). When stratified by physical activity level, seven participants with low activity, nine with moderate activity, and one with high activity reported experiencing EOP. Fluid intake also varied among those reporting edema; four participants (23.5%) reported consuming 20-39.9 oz/day, four (23.5%) reported 40-59.9 oz/day, and nine (52.9%) reported intake exceeding 60 oz/day (Table 2).

**Table 2:** Maternal characteristics and potential risk factors for pregnancy-related edema Abbreviations: CHF, Congestive Heart Failure; CKD, Chronic Kidney Disease; DM, Diabetes Mellitus; DVT, Deep Vein Thrombosis; HELLP, Hemolysis, Elevated Liver Enzymes, and Low Platelet Count; ICP, Intrahepatic Cholestasis of Pregnancy; VBAC, Vaginal Birth After Cesarean

Variable	All Patients (n = 52)	Pregnancy-Related Edema (n = 18)	No Edema (n = 34)
Age (mean, range in years)	30 (21–39)	32.05 (23–39)	28.76 (21–37)
Gravidity (mean, range)	2.5 (1–8)	2.71 (1–6)	2.32 (1–8)
Parity (mean, range)	2.1 (1–6)	2.29 (1–6)	2.02 (1–5)
Gestational age at delivery (weeks and days)	35w2d–41w1d (38w2d)	35w2d–41w1d (38w2d)	35w3d–40w6d (38w1d)
Vaginal delivery	31 (59.6%)	8 (47.1%)	23 (67.5%)
VBAC	1 (1.9%)	0	1 (2.9%)
C-section	15 (28.8%)	7 (41.2%)	7 (22.9%)
Emergent C-section	5 (9.6%)	2 (11.8%)	3 (8.6%)
Gestational DM	5 (9.6%)	2 (11.8%)	3 (8.6%)
Type 2 DM	1 (1.9%)	0	1 (2.9%)
Chronic hypertension	2 (3.8%)	1 (5.9%)	1 (2.9)
Gestational hypertension	8 (15.4%)	4 (23.5%)	4 (11.4%)
Pre-eclampsia	4 (7.7%)	2 (11.8%)	2 (3.8%)
Eclampsia, CHF, CKD, HELLP, twin pregnancy	0	0	0
ICP	1 (1.9%)	0	1 (2.9%)
Obesity	11 (21.2%)	8 (47.1%)	3 (8.6%)
Placenta previa	2 (3.8%)	0	2 (3.8%)
Polyhydramnios	1 (1.9%)	0	1 (1.9%)
DVT/fracture	0	0	0
Lower-extremity injury	3 (5.8%)	2 (Achilles rupture, 11.8)	1 (compartment syndrome, 2.9%)
Activity level low	13 (25%)	7 (41.2%)	6 (17.1%)
Activity level medium	30 (57.7%)	9 (52.9%)	21 (60.0%)
Activity level high	9 (17.3%)	1 (5.9%)	8 (22.9%)
Water intake 0–19.9 oz	0	0	0
Water intake 20–39.9 oz	10 (19.2%)	4 (23.5%)	6 (17.1%)
Water intake 40–59.9 oz	15 (28.8%)	4 (23.5%)	11 (31.4%)
Water intake >60 oz	27 (51.9%)	9 (52.9%)	18 (51.4%)

Participants with EOP rated their discomfort at an average of 3.8 on a 10-point scale (standard deviation 2.6) and the severity of EOP at an average of 4.5 on a 10-point scale (standard deviation 2.3). They reported trying elevation (n = 12, 70.6%), compression (n = 8, 47.1%), and massage (n = 3, 17.6%) to relieve edema symptoms. The most common pain symptoms included back pain (n = 12, 70.6%), pelvic pain (n = 5, 29.4%), and carpal tunnel pain (n = 5, 29.4%).

Logistic regression analysis was performed to understand the relationship between the variables and EOP. We observed a statistically significant association between obesity and EOP. During pregnancy, individuals with obesity had a significantly higher likelihood of developing edema, with an odds ratio of 9.48 (p = 0.003, α < 0.05). A similar association was found in the postpartum period, with obesity being associated with increased odds of postpartum edema (odds ratio = 8.5, p = 0.004, α < 0.05). While other variables, including hypertensive disorders, physical activity levels, and fluid intake, were associated with increased odds of edema, these associations did not reach statistical significance in this sample (Table 3). These trends may indicate potential risk factors for the development of EOP; however, larger studies are warranted to validate these findings and clarify their clinical relevance.

**Table 3:** Association between edema of pregnancy and various risk factors * p<0.05 Abbreviations: DM, Diabetes Mellitus; DVT, Deep Vein Thrombosis; HELLP, Hemolysis, Elevated Liver Enzymes, and Low Platelet Count

Risk Factor	Pregnancy Edema (p-values)	Pregnancy Edema Odds Ratios (OR)	Postpartum Edema (p-values)	Postpartum Edema Odds Ratios (OR)
Type 2 DM	0.99	3.47	0.99	4.59
Gestational DM	0.71	1.42	0.71	0.65
Chronic hypertension	0.60	2.12	0.47	2.84
Gestational hypertension	0.26	2.38	0.46	1.80
Pre-eclampsia	0.46	2.13	0.31	2.91
Obesity	0.003*	9.48	0.004*	8.5
Intrahepatic cholestasis	0.99	3.47	0.99	4.59
Placenta previa	0.99	1.24	0.99	1.64
Ligament injury	0.23	4.53	0.78	1.38
Water intake (20–39.9 oz)	0.58	1.48	0.08	3.67
Water intake (40–59.9 oz)	0.55	0.67	0.17	0.32
Water intake (>60 oz)	0.91	1.06	0.86	0.90
Low physical activity (0–59 minutes per week)	0.06	3.28	0.71	1.28
Medium physical activity (60–119 minutes per week)	0.62	0.75	0.56	1.45
High physical activity (>120 minutes per week)	0.15	0.21	0.26	0.28

## Discussion

In this study, 32.7% of postpartum participants reported experiencing EOP, underscoring its prevalence and clinical relevance in the perinatal period. Among the risk factors evaluated, obesity demonstrated the strongest association with EOP in our sample. This aligns with existing literature linking maternal obesity to adverse obstetric outcomes, including gestational diabetes, hypertensive disorders, and thromboembolic events [13]. Interestingly, prior studies have reported conflicting findings. For example, Ochalek et al. found no significant association between BMI and pregnancy-related edema [7], highlighting the need for further investigation into this relationship.

Physical activity emerged as another important variable. Previous research has identified decreased activity as a contributor to fluid retention during pregnancy [7,14-16]. Our findings were consistent with this, showing that low physical activity levels were associated with an increased likelihood of EOP (OR = 3.28, p = 0.06), approaching statistical significance. Conversely, participants reporting higher levels of physical activity exhibited a potentially protective trend (OR = 0.21, p = 0.15). Although these associations did not reach statistical significance, the observed patterns suggest clinically relevant trends that merit exploration in larger, adequately powered studies.

Hypertensive disorders, including chronic hypertension, gestational hypertension, and preeclampsia, have been previously linked to increased fluid retention during pregnancy [7]. In our analysis, these conditions were associated with increased odds of edema (ORs ranging from 2.12 to 2.38), though none of them achieved statistical significance. These findings may reflect the limited sample size or insufficient numbers within diagnostic subgroups, reducing the power to detect meaningful associations.

Water intake also demonstrated variable associations with EOP. Notably, extremely low fluid consumption (<20 oz/day) was linked to the presence of edema, though the odds ratio for this group was reported as 0, likely reflecting a lack of variation between comparison groups and limited statistical reliability. Moderate fluid intake (40-59.9 oz/day) showed a trend toward reduced odds of edema (OR = 0.67), supporting the hypothesis that adequate hydration may contribute to improved fluid balance in pregnancy.

Several other comorbid conditions, including type 1 diabetes, eclampsia, congestive heart failure, chronic kidney disease, HELLP syndrome, twin pregnancy, and DVT, returned odds ratios of 0. These results most likely reflect a lack of observed cases in our sample rather than the absence of association. Consequently, no definitive conclusions can be drawn regarding their relationship to EOP based on this dataset.

Collectively, these findings underscore the multifactorial etiology of EOP, with obesity and physical activity emerging as potentially modifiable risk factors. The observed trends are consistent with prior literature and highlight areas for future research. Larger, prospective cohort studies are needed to clarify the complex interactions between maternal characteristics, comorbidities, and lifestyle behaviors in the development and progression of pregnancy-related edema.

This study has several limitations. First, the use of a paper-based, self-administered survey introduces the possibility of recall bias, particularly given the physical and emotional demands of the early postpartum period. Social desirability bias [17] and the availability heuristic [18] may also have influenced how participants reported symptoms, especially in the absence of standardized symptom grading.

Second, the relatively small sample size (n = 52) limits the statistical power of the analysis, increasing the risk of Type II errors and reducing confidence in subgroup comparisons. The study’s setting, limited to two Midwestern hospitals, further constrains the generalizability of the findings to broader obstetric populations.

The cross-sectional design provides only a single time-point assessment, precluding inferences about causality or symptom trajectory. Additionally, unmeasured confounders such as pre-existing conditions, medications, dietary patterns, or genetic factors were not accounted for, which may have influenced the results. The exclusive reliance on subjective, self-reported symptoms rather than objective clinical assessments (e.g., ultrasound, limb circumference, or bioimpedance) may have led to under- or over-reporting of edema. Lastly, edema is a dynamic condition influenced by daily fluctuations in activity and fluid intake, which may not have been fully captured through a retrospective questionnaire.

## Conclusions

This study has several limitations. First, the relatively small sample size limits statistical power and may reduce the ability to detect modest but clinically meaningful associations. Second, the cross-sectional design precludes assessment of temporal relationships and limits causal inference. Third, reliance on self-reported symptoms introduces the potential for recall bias and subjective misclassification. Fourth, the study population was drawn from only two hospitals in the Midwest, which may limit generalizability to other geographic regions or healthcare settings. Finally, the absence of objective measures of edema severity, such as limb circumference measurements or ultrasound-based assessments, restricts the precision of phenotypic characterization.

Regardless, our findings suggest that lower-extremity edema in pregnancy affects nearly one-third of postpartum patients and may be influenced by modifiable risk factors such as obesity and physical activity. Although not all observed associations reached statistical significance, several demonstrated meaningful clinical trends that warrant further investigation. An improved understanding of these risk factors is essential for developing targeted interventions and providing anticipatory guidance for affected individuals. Future research should prioritize longitudinal, multicenter studies that incorporate both objective and subjective measures to better characterize the natural history, risk profile, and optimal management of EOP.

## Appendices

Appendix 1

Study questionnaire 

1. Age: _______ 

2. How many pregnancies have you had? Include all miscarriages/abortions. ___________ 

3. How many living children do you have? ___________ 

4. What gestational age were you when you delivered? _______weeks _______ days  

5. How did you deliver? 

a) Vaginal       

b) Vaginal after cesarean section 

c) Planned cesarean   

d) Emergent cesarean  

6. Did you have any of the following pregnancy complications or comorbid conditions? Select all that apply. 

a) Type I Diabetes  

b) Type II Diabetes  

c) Gestational Diabetes  

d) Hypertension  

e) Gestational Hypertension  

f) Preeclampsia  

g) Eclampsia  

h) Congestive Heart Failure 

i) Chronic Kidney Disease 

j) HELLP Syndrome  

h) Intrahepatic Cholestasis 

i) Twin pregnancy 

j) Obesity (pre-pregnancy BMI (Body Mass Index) >30) 

k) Other ___________________________ 

7. Have you had any issues with your lower extremities in the past year?  

a) DVT (blood clot)  

b) Fractured bone- if so, what bone? ______________________ 

c) Ligamentous injury (including ankle sprain)  

d) Other _____________________ 

8. Did you have edema during this pregnancy?  

a) Yes  

b) No 

9. If you answered yes to 8, how would you rate your edema on a scale of 1-10, with 10 being most severe? _______/10  

10. If you answered yes to 8, how would you rate the discomfort you experienced from your edema on a scale of 1-10, with 10 being the most pain? _____/10  

11. If you answered yes to 8, what did you do to help reduce your edema during your pregnancy?  

a) Compression stockings/wraps 

b) Massage therapy  

c) Elevation  

d) Osteopathic manipulative therapy  

e) Other ___________________ 

12. Do you have any postpartum edema?  

a) Yes  

b) No  

13. How would you rate your activity level during your pregnancy on average?  

a) Low (0-59 minutes per week)  

b) Medium (60-119 minutes per week)  

c) High (more than 120 minutes per week)  

14. How much fluid do you drink in a day? Include all water, coffee, tea, etc. For reference, a standard water bottle is 16.9 oz, and a quart is 32 oz. 

a) 0-19.9 oz  

b) 20-39.9 oz  

c) 40-59.9 oz  

d) >60 oz  

15. Did you have any other symptoms related to your pregnancy (select all that apply)?  

a) Headaches  

b) Neck pain  

c) Back pain  

d) Pubic symphysis/pelvic pain  

e) Carpal Tunnel  

f) Constipation        
